# Recurrent group G *Streptococcus* bacteremia: A case report and literature review

**DOI:** 10.1002/ccr3.6162

**Published:** 2022-08-08

**Authors:** Haider Ghazanfar, Zaheer Qureshi, Harika Kalangi, Subhan Ata, Abhilasha Jyala, Esther Arguello Perez

**Affiliations:** ^1^ BronxCare Health System, Internal Medicine 1650 Selwyn Avenue Bronx New York USA; ^2^ BronxCare Health System, Infectious Diseases 1650 Selwyn Avenue Bronx New York USA

**Keywords:** bacteremia, morbidity, mortality, recurrent, risk factor, *Streptococcus* G

## Abstract

Streptococci group G is an important opportunistic pathogen and causes a wide variety of infections, including pharyngitis, skin and soft tissue infections, bacteremia, endocarditis, septic arthritis, intra‐abdominal infections, meningitis, and streptococcal toxic shock‐like syndrome. As a result, we discuss an interesting case of recurrent group G streptococcal bacteremia in a 68‐year‐old man presenting with altered mental status. We also discuss the risk factors, etiology, pathophysiology, diagnosis, and treatment of group G streptococcal bacteremia.

## 1 INTRODUCTION

Species of *Streptococcus* are classified based on their hemolytic properties. Alpha‐hemolytic, beta‐hemolytic, and gamma‐hemolytic species. Beta‐hemolytic streptococci are further classified by Lancefield grouping, a serotype classification. The 21 described serotypes are named Lancefield groups A to W. Among group G streptococci (GGS), *Streptococcus dysgalactiae* is the predominant species encountered, particularly in human disease.^1^ GGS has been identified as part of the normal flora of human skin, oral cavity, nasopharynx, gastrointestinal tract, and vagina. It has been widely accepted that group G species generally cause opportunistic and nosocomial infections in patients with the underlying medical condition.^1^ This report describes a patient diagnosed with recurrent group G *Streptococcal* bacteremia.

## 2 CASE PRESENTATION

Our patient is a 68‐year‐old man brought to the emergency department with altered mental status and acute respiratory distress since evening. As per a family member, he was in his usual state of health until the morning of the presentation day. His past medical history was significant for hypertension, hyperlipidemia, chronic obstructive pulmonary disease (COPD), history of deep venous thrombosis and pulmonary embolism (IVC filter placed in 1986; currently on Coumadin), obstructive sleep apnea (on continuous positive airway pressure therapy), and nonischemic heart failure with reduced ejection fraction. He also had a group G streptococcal bacteremia due to the right lower extremity cellulitis 2 years ago. His past surgical history was significant for cholecystectomy, appendectomy, right total knee replacement, gastric bypass surgery, and right varicose vein surgery. He quit smoking and drinking 22 years back.

In the emergency department, he was confused and hypoxic with oxygen saturation of 70% on room air. He had a temperature of 99.3 degrees Fahrenheit, a heart rate of 117 beats per minute, a respiratory rate of 28 breaths per minute, and a blood pressure of 135/102 mm Hg. His lung examination revealed moderate wheezing bilaterally and fine bilateral basal crackles. He had venous stasis and chronic bilateral leg ulcers along with pedal edema. He was not alert and oriented to time, place, and person. He was treated with an albuterol nebulizer, supplemental oxygen, intravenous magnesium sulfate 2 gm, intravenous furosemide 40 mg, intravenous methylprednisolone, and intravenous naloxone 0.6 mg. His mental status improved after the initial treatment, and he was placed on bi‐level positive airway pressure (BiPAP).

Initial laboratory analysis is presented in Table 1.

**TABLE 1:** Patient's initial laboratory results

Investigations	Results
Complete blood count	
Red blood cell count	3.45 (4.50–5.90 MIL/μl)
Hemoglobin	10.8 (12.0–16.0 g/dl)
Hematocrit	32.7 (42.0–51.0%)
Platelet	98 (150–400 k/μl)
White blood cell count	5.1 (4.8–10.8 k/μl)
Neutrophil %	89.1 (40.0–70.0%)
Chemistry	
Sodium	139 (135–145 mEq/L)
Potassium	4.7 (3.5–5.0 mEq/L)
Bicarbonate	28 (24–30 mEq/L)
Blood urea nitrogen	16 (8–26 mg/dl)
Creatinine	0.6 (0.5–1.5 mg/dl)
Phosphorus	3.9 (2.4–4.5 mg/dl)
Magnesium	1.9 (1.5–2.7 mg/dl)

**FIGURE 1:**
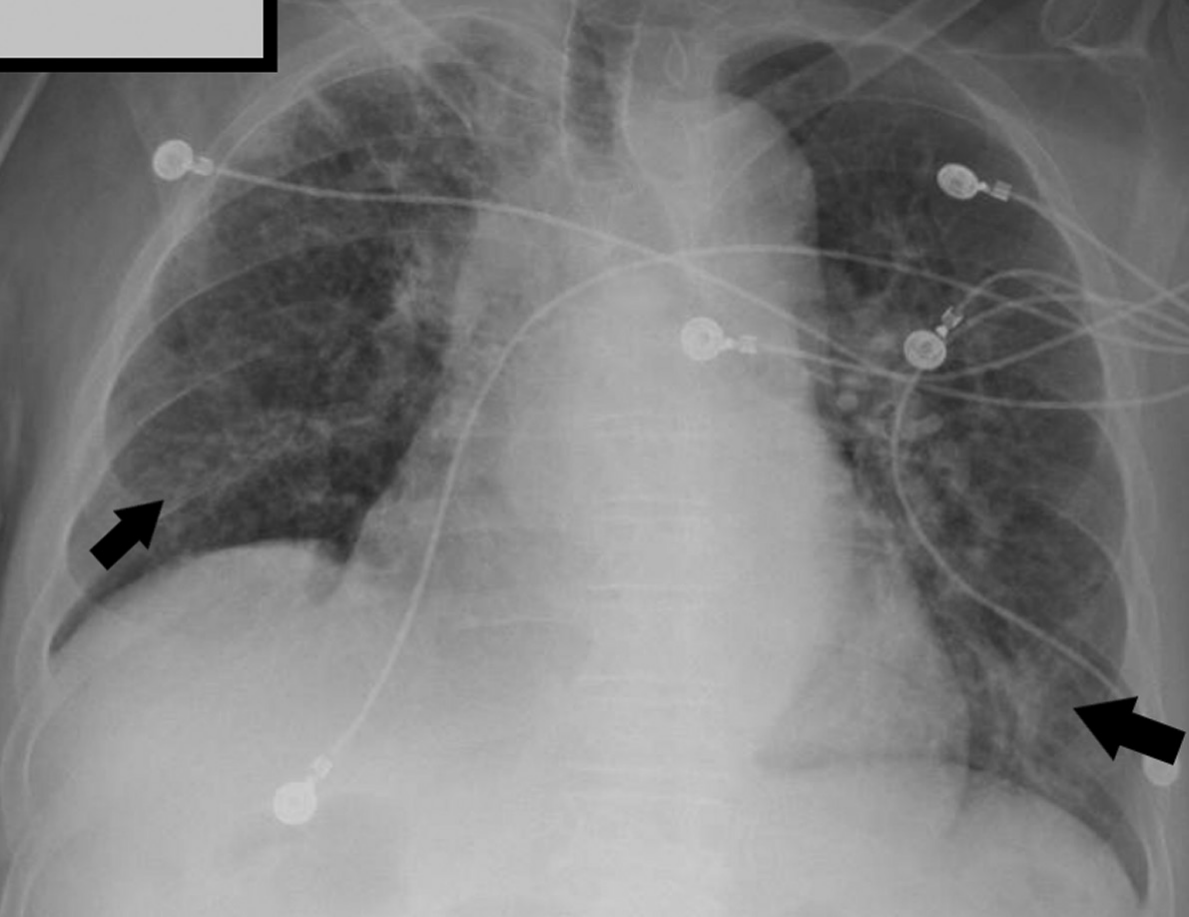
Chest X‐ray showing bilateral perihilar interstitial opacities

His electrocardiogram (ECG) revealed atrial fibrillation with rapid ventricular response. Chest X‐ray showed bilateral perihilar interstitial opacities, as shown in Figure 1.

The patient was admitted to the intensive care unit (ICU) for the management of COPD exacerbation, pneumonia, atrial fibrillation with rapid ventricular response, congestive heart failure, exacerbation, and encephalopathy. One set of blood cultures was sent. The patient was started on empirical antibiotic therapy for community‐acquired pneumonia with ceftriaxone and azithromycin. Supplemental oxygen and intravenous methyl‐prednisone were started for COPD exacerbation. Preliminary blood culture was positive for gram‐positive cocci, so vancomycin was added to the regimen. Blood culture subsequently revealed group G *Streptococcus*. Based on the antibiogram, therapy was switched to ceftriaxone as GGS was pan‐sensitive. The patient was then switched to oral cefuroxime to complete 14 days of antibiotics. Repeat blood cultures were negative. Transthoracic echocardiogram showed no evidence of valvular vegetation. The patient improved with the treatment and was discharged in a stable condition.

## 3 DISCUSSION

Group G streptococci is known to cause many infections, including pharyngitis, skin and soft tissue infections, bacteremia, endocarditis, septic arthritis, intra‐abdominal infections, meningitis, and streptococcal toxic shock‐like syndrome.^2^, ^3^ Skin and soft tissue infections have been the most common manifestations of infection by these organisms. Group G bloodstream infection incidence has been reported from 3.2 to 5.5 per 100,000 populations.^1^, ^4^, ^5^ Several studies have reported an increased disease incidence due to GGS.^6^, ^7^ Mortality has been reported between 8 and 21% in different studies population.^1^, ^3^, ^4^, ^8^

Predisposing factors for bacteremia are cancer, uncontrolled diabetes mellitus, chronic heart or lung diseases, immunodeficiency or alcohol abuse, cirrhosis, bone and joint disease, and illicit drug abuse.^6^, ^8^, ^9^, ^10^ Our patient had chronic heart and lung diseases. The majority of bacteremia has been community‐acquired.^9^, ^10^

For group G streptococcal bacteremia, the most frequent portal of entry was the skin, usually in cases with pre‐existing edema due to previous surgical removal, irradiation, tumor infiltration of lymph nodes, or chronic venous insufficiency. According to a retrospective study done in Israel from 1989 through 2000, of the 94 episodes of GGS bacteremia, 6 were recurrent infections.^11^ In our patient, we believe the cause for the recurrent GGS bacteremia was the chronic venous stasis ulcers on both legs.

Virulence factors of GGS include adhesins, proteases, and toxins. GGS targets fibronectin, which is found on the epithelial cells present in the upper respiratory tract. This is a critical step in developing an infection as it enables the GGS to attach and infect the cell. The M protein virulence factor can bind to some serum proteins, prevent phagocytosis, and disrupt the coagulation system, which facilitates the spread of infection.^12^ GGS produces the toxin streptolysin 0 and streptolysin S, which are involved in necrotizing soft tissue infection.^13^ Transmission may occur from person to person by respiratory droplets or skin contacts.^14^ Invasive infections are rare and have been reported with underlying diseases such as vascular/lymphatic compromise, diabetes, cardiovascular disease, malignancy, advanced age, and alcohol use.^5^, ^8^

Patients with GGS identification on blood, synovial, or cerebrospinal fluid should receive treatment without delay as GGS contamination in these compartments is rare. Identification on pharyngeal or skin wound cultures is less specific due to the natural site of colonization.^15^ Group G *Streptococcus* is susceptible to beta‐lactam antibiotics, and penicillin is the treatment of choice. Most of the GGS species are susceptible to beta‐lactam antibiotics. Penicillin remains the drug of choice for treating GGS infections.^16^ Alternative regimens include cephalosporins and the addition of clindamycin if there is a concern for streptococcal toxic shock syndrome.^17^ Unlike GAS, GGS does not cause rheumatic fever and requires less aggressive treatment for eradication.^18^ For patients with a history of severe hypersensitivity reactions to beta‐lactam antibiotics, vancomycin may be used.^16^, ^19^

## 4 CONCLUSION

We conclude that invasive group G *Streptococcus* infection increases incidence, especially in the elderly population with underlying chronic diseases. Physicians need to be aware that chronic venous stasis ulceration can lead to recurrent GGS bacteremia. Penicillins are classically considered a drug of choice, and alternative regimens include third‐generation cephalosporins, such as ceftriaxone or cefotaxime. The addition of clindamycin should be considered if there is a concern for streptococcal toxic shock syndrome.

## AUTHOR CONTRIBUTIONS

H. Ghazanfar, Z. Qureshi, H. Kalangi, S. Ata, and A. Jyala have made substantial contributions to conception and design. H. Ghazanfar, Z. Qureshi, H. Kalangi, S. Ata, A. Jyala, and E. Perez drafted the manuscript and revised it critically for important intellectual content. Z. Qureshi agreed to be accountable for all aspects of the work in ensuring that questions related to the accuracy or integrity of any part of the work are appropriately investigated and resolved.

## FUNDING INFORMATION

None.

## CONFLICT OF INTEREST

None.

## CONSENT

Written informed consent was obtained from the patient to publish this report in accordance with the journal's patient consent policy.

## Data Availability

The data that support the findings of this study are available on request from the corresponding author. As the study includes medical records of an individual person, the data are not publicly available due to privacy or ethical restrictions.
